# SCORE: Shared care of Colorectal cancer survivors: protocol for a randomised controlled trial

**DOI:** 10.1186/s13063-017-2245-4

**Published:** 2017-10-30

**Authors:** Michael Jefford, Jon Emery, Eva Grunfeld, Andrew Martin, Paula Rodger, Alexandra M. Murray, Richard De Abreu Lourenco, Alexander Heriot, Jo Phipps-Nelson, Lisa Guccione, Dorothy King, Karolina Lisy, Niall Tebbutt, Adele Burgess, Ian Faragher, Rodney Woods, Penelope Schofield

**Affiliations:** 1 Department of Cancer Experiences Research, Peter MacCallum Cancer Centre, Melbourne, VIC Australia; 2 Sir Peter MacCallum Department of Oncology, Faculty of Medicine, Dentistry and Health Sciences, The University of Melbourne, Melbourne, VIC Australia; 3 Division of Cancer Medicine, Peter MacCallum Cancer Centre, 305 Grattan Street, Melbourne, VIC 3000 Australia; 4 Department of General Practice and Centre for Cancer Research, University of Melbourne, Victorian Comprehensive Cancer Centre, Melbourne, VIC Australia; 5 Ontario Institute for Cancer Research, Toronto, ON Canada; 6 Department of Family and Community Medicine, University of Toronto, Toronto, ON Canada; 7 National Health and Medical Research Council (NHMRC) Clinical Trials Centre, University of Sydney, Sydney, NSW Australia; 8 Centre for Health Economics Research and Evaluation, University of Technology Sydney, Sydney, NSW Australia; 9 Division of Cancer Surgery, Peter MacCallum Cancer Centre, Melbourne, VIC Australia; 10 Psychology Department, School of Health and Biomedical Sciences, RMIT University, Melbourne, VIC Australia; 11 Department of Medical Oncology, Olivia Newton-John Cancer Wellness and Research Centre, Austin Health, Heidelberg, VIC Australia; 12 Colorectal Surgery Unit, Austin Health, Heidelberg, VIC Australia; 13 Colorectal Surgery, Western Health, Footscray, VIC Australia; 14 Colorectal Surgery Unit, St Vincent’s Hospital, Fitzroy, VIC Australia; 15 Department of Psychology, School of Health Sciences, Faculty of Health, Arts and Design, Swinburne University of Technology, Heidelberg, VIC Australia

**Keywords:** Colorectal cancer, Survivorship, Follow-up, Shared care, Primary care, Models of care, Protocol

## Abstract

**Background:**

Colorectal cancer (CRC) is the most common cancer affecting both men and women. Survivors of CRC often experience various physical and psychological effects arising from CRC and its treatment. These effects may last for many years and adversely affect QoL, and they may not be adequately addressed by standard specialist-based follow-up. Optimal management of these effects should harness the expertise of both primary care and specialist care. Shared models of care (involving both the patient’s primary care physician [PCP] and specialist) have the potential to better support survivors and enhance health system efficiency.

**Methods/design:**

SCORE (Shared care of Colorectal cancer survivors) is a multisite randomised controlled trial designed to optimise and operationalise a shared care model for survivors of CRC, to evaluate the acceptability of the intervention and study processes, and to collect preliminary data regarding the effects of shared care compared with usual care on a range of patient-reported outcomes. The primary outcome is QoL measured using the European Organisation for Research and Treatment of Cancer QLQ-C30 questionnaire. Secondary outcomes are satisfaction with care, unmet needs, continuity of care and health resource use. The shared care model involves replacement of two routine specialist follow-up visits with PCP visits, as well as the provision of a tailored survivorship care plan and a survivorship booklet and DVD for CRC survivors. All consenting patients will be randomised 1:1 to either shared care or usual care and will complete questionnaires at three time points over a 12-month period (baseline and at 6 and 12 months). Health care resource use data will also be collected and used to evaluate costs.

**Discussion:**

The evaluation and implementation of models of care that are responsive to the holistic needs of cancer survivors while reducing the burden on acute care settings is an international priority. Shared care between specialists and PCPs has the potential to enhance patient care and outcomes for CRC survivors while offering improvements in health care resource efficiency. If the findings of the present study show that the shared care intervention is acceptable and feasible for CRC survivors, the intervention may be readily expanded to other groups of cancer survivors.

**Trial registration:**

Australian New Zealand Clinical Trials Registry, ACTRN12617000004369p. Registered on 3 January 2017; protocol version 4 approved 24 February 2017.

**Electronic supplementary material:**

The online version of this article (doi:10.1186/s13063-017-2245-4) contains supplementary material, which is available to authorized users.

## Background

### Colorectal cancer: high burden of illness

Colorectal cancer (CRC; also known as *bowel cancer*) is the most common cancer affecting both men and women (excluding non-melanoma skin cancers) [1]. Although it is the second highest cause of death from cancer (after lung cancer), many people are long-term survivors. Survivors of CRC represent the third largest group of long-term cancer survivors in the Western world (after survivors of breast and prostate cancer) [2]. In the United States, an estimated 1.4 million people have a personal history of CRC [2]. It is expected that the number of people affected by and surviving CRC will rise significantly over the next 10 years. US data suggest an almost 24% increase in the number of CRC survivors between 2016 and 2026 [2].

Survivors report a broad range of consequences from CRC and its treatment, including persistent side effects, such as fatigue [3–8]; bowel, urinary and sexual dysfunction [3, 4, 7–18]; and neuropathy [6, 10, 19]. CRC survivors may experience elevated levels of psychological distress and depression [7, 16, 18, 20–23]. Fear of cancer recurrence is common [4, 7, 8, 24, 25]. Unsurprisingly, QoL among CRC survivors may be impaired compared with the general population [3, 5, 8, 9, 12, 14, 22, 26].

In our recent SurvivorCare study [27], a randomised controlled trial (RCT) investigating the impact of a supportive care intervention for CRC survivors, when compared with members of the general Australian population, CRC survivors around the time of treatment completion reported higher levels of fatigue, pain, nausea and vomiting, appetite loss, diarrhoea, constipation and financial problems [28]. They also reported lower levels of role, cognitive and social functioning. Commonly reported issues included frequent urination (67%), problems with taste (56%) and worries about future health (71%). In survivors with a stoma, 52% reported frequent bag changes, 66% reported flatulence and 61% reported some degree of leakage of stools. For survivors without a stoma, 68% reported flatulence, 36% had some degree of leakage of stools and 70% reported frequent daily bowel movements. Sixty-five percent of men reported some degree of impotence [28].

Similar findings were seen in a study of over 21,000 CRC survivors in England who were 12–36 months from diagnosis, again underscoring that CRC survivors have ongoing, unresolved symptom issues [12]. A further study of CRC survivors reported that addressing emotional problems during follow-up was important for patients but was commonly neglected [11].

Survivors frequently report unmet needs, including for more comprehensive, coordinated care; for more information; and for psychological support [15, 25, 28–31]. Again, in our SurvivorCare study, the most commonly endorsed need was ‘I need to know that all my doctors talk to each other to coordinate my care’ (68% endorsement) [28].

### Inadequacies of current follow-up care after completing treatment

The Institute of Medicine (IOM), in its report ‘From Cancer Patient to Cancer Survivor: Lost in Transition’, asserts, ‘The transition from active treatment to post-treatment care is critical to long-term health. If care is not planned and coordinated, cancer survivors are left without knowledge of their heightened risks and a follow-up plan of action’ [32]. After completing treatment, most patients have ongoing follow-up with a cancer specialist [32, 33]. Despite the known complex and distressing concerns survivors of CRC face, follow-up guidelines are focussed largely on strategies to detect recurrence or possible second cancers [34, 35]. The IOM report adds, ‘Notably absent is guidance regarding the functional sequelae that may follow surgical interventions (e.g., colostomy, bowel dysfunction, sexual dysfunction)’ [32]. Current models of care are inadequate, with limited attention given to supportive care issues, preventive care and management of comorbid illness [32, 34, 36]. Additionally, current models of specialist-based care are expensive and likely unsustainable [32, 37]. There is a mismatch between the availability of cancer services and the growing number of survivors [37]. Nevertheless, very little research has been focussed on the post-treatment care of CRC survivors [38].

### Alternative models of patient follow-up

Grunfeld led the first RCTs comparing follow-up with primary care physicians (PCPs) versus specialist-based care, for breast cancer survivors [39–44]. These studies showed that disease outcomes [41, 43] and patient QoL [39] were similar, though PCP-based care was associated with improved patient satisfaction [39] and lower costs, for both patients and health services [40]. In a single, Australia-based RCT, researchers evaluated PCP follow-up versus specialist follow-up for people with CRC [45]. No compelling evidence for a difference was found.

These studies had a relatively narrow focus, primarily on the detection of cancer recurrence. Recommended survivorship care should also determine survivors’ concerns, attend to treatment side effects and comorbid illness, and ensure support, as necessary.

### Shared care between primary care physicians and specialists

Despite the results of the above-described RCTs, PCP-based follow-up has not been widely adopted. Our own work involving survivors, PCPs, surgeons and oncologists indicated strong endorsement that PCPs be involved in the ongoing care of CRC survivors and provided with information to facilitate care [46].

The vast majority of cancer survivors have coexisting illness [47, 48]. Survivors who have follow-up involving primary care are more likely to receive preventive interventions and have appropriate management of comorbid illness [36].

Models of post-treatment care should address the holistic health care needs of cancer survivors with optimal cancer-specific follow-up, management of comorbid conditions, and general preventive health care [49, 50]. These models of care should optimise the expertise of different providers for the benefit of patients and efficiency of the health care system.

An alternative to the current model of oncology-based survivorship care is a shared care model, harnessing the expertise of both PCPs and specialists [51–53]. Shared care is widely used in antenatal care and in the management of patients with conditions such as asthma, diabetes and ischaemic heart disease. Few studies have evaluated shared care with cancer survivors. In our randomised phase II study involving men with prostate cancer, ProCare [54], we found distress levels, QoL and satisfaction were similar in both groups; shared care was preferred by men who had experienced it, and the shared care model was less costly [55].

### Principles underpinning a novel shared care model of follow-up

The Shared care of Colorectal cancer survivors (SCORE) intervention is underpinned by considerations in the Chronic Care Model (CCM) [56] and recommendations from the IOM regarding post-treatment care [32].

Studies suggest that redesigning care using the CCM leads to improved patient care and better health outcomes [57, 58]. As recommended by the CCM, SCORE is focussed on patients, professionals and the organisation of care. Key elements include structured clinical follow-up, reminders and education for professionals, and patient education and self-management support. These strategies aim to improve continuity and coordination of care and enhance patient outcomes [59].

In its report ‘From Cancer Patient to Cancer Survivor: Lost in Transition’, the IOM outlined four essential components of post-treatment survivorship care: (1) prevention of recurrent and new cancers, and of other late effects; (2) surveillance for cancer spread, recurrence or second cancers, as well as assessment of medical and psychosocial late effects; (3) intervention for consequences of cancer and its treatment; and (4) coordination between specialists and primary care providers to ensure that all of the survivor’s health needs are met [32]. A recent review of guidelines regarding follow-up of CRC survivors highlighted that most are focussed on the detection of cancer recurrence and assessment of the medical consequences of treatment, with little attention placed on identifying and responding to other key unmet needs [34]. The aim of SCORE is to provide more comprehensive, holistic care. The principles that inform the SCORE model are described in the subsections that follow.

#### Communication between specialists and PCPs

A current major barrier is coordination of care between specialists and PCPs. Timely and systematic communication between specialists and PCPs is urgently required to clarify the roles and responsibilities of all, including the person with cancer [60]. We previously showed that faxing standardised information to PCPs about a patient’s chemotherapy regimen improved confidence of PCPs in managing adverse effects of treatment and increased satisfaction with shared care [61]. PCPs will be provided with timely patient-specific information and clinical guidance.

#### Promotion of patient involvement and engagement

The majority of Australian patients with cancer want to be involved with decision-making and wish to participate in strategies to remain well [62]. Involving patients with chronic diseases in their disease management results in better communication with physicians, improved self-reported health and reduced health distress, fewer hospitalisations, and reduced health costs [63, 64]. A systematic review of patient activation approaches has shown these strategies can alter the content of consultations and improve the identification of patients’ concerns [65]. Approaches that allow patients to list and share their concerns with their doctors, particularly if linked to practitioner interventions, showed particular promise. Patients will have a mechanism to identify and discuss concerns with their PCP.

#### Tailoring to specific needs of individual patients

Cancer survivors have individualised needs [28, 66]. Therefore, interventions need to be systematically tailored to each individual. Authors of a review of tailored versus standardised information interventions in the health promotion area found that tailored interventions were significantly more effective in promoting health behaviour outcomes [67]. SCORE will enable both patients (survivors) and PCPs to identify issues of concern to the individual person.

### Objectives and trial design

#### Objectives

The objectives of the trial are to operationalise and optimise the shared care intervention; establish acceptability of the intervention, randomisation, outcome measures and study processes; obtain preliminary estimates of effects on patient-reported outcome measures and health care resource use; and to confirm the appropriateness of expansion to a definitive phase III trial.

#### Hypotheses

We hypothesise that, relative to usual care, shared care will be an acceptable model of follow-up with the potential to address care needs more efficiently than standard care.

#### Trial design

**Fig. 1:**
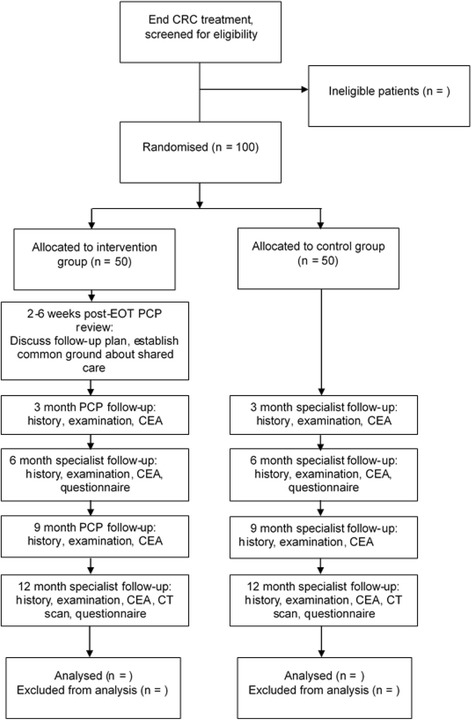
Flow diagram of patient recruitment and study conduct. *CEA* Carcinoembryonic antigen, *CRC* Colorectal cancer, *CT* Computed tomography, *EOT* End of treatment, *PCP* Primary care physician

This is a randomised, parallel group trial in which patients with CRC undergoing primary treatment with curative intent will be allocated to receive either standard specialist-based follow-up care or shared follow-up care between their specialist and PCP. Clinical reviews will occur at 3, 6, 9 and 12 months. Patient self-reported measures will be done at baseline and at 6 and 12 months. The trial will be conducted in accordance with good clinical practice guidelines and the Declaration of Helsinki. The protocol is in line with the Standard Protocol Items: Recommendations for Interventional Trials guidelines [68] and the Consolidated Standards for Reporting of Trials guidelines [69] (Fig. 1, study flow diagram) (Additional file 1). The trial is registered with the Australian New Zealand Clinical Trials Registry (number 12617000004369p).

## Methods/design

### Participants, interventions and outcomes

#### Study setting

The study will be conducted at the Peter MacCallum Cancer Centre, Royal Melbourne Hospital, Western Health, St Vincent’s Hospital and Austin Health, Melbourne, Victoria, Australia. Each site treats a considerable number of people with CRC.

#### Eligibility criteria

*Inclusion:* To be eligible, patients must (1) have a histologically confirmed diagnosis of colon or rectal cancer, (2) have stage I–III disease, (3) have completed treatment with curative intent with surgery with or without radiation with or without chemotherapy within 2 months, (4) be over 18 years of age, (5) be able to understand English, and (6) have a PCP who is willing to participate in the study.

*Exclusion:* Exclusion criteria are patients (1) with demonstrated cognitive or psychological difficulties that would preclude study participation as defined by the treatment team, (2) who are too unwell to participate in the study as determined by the patient’s treatment team, (3) who have received treatment for a prior cancer (excluding non-melanoma skin cancer), and (4) who have a PCP who is already participating in the study (to avoid contamination between randomisation groups).

*Withdrawal:* Participants will be withdrawn if (1) they have cancer recurrence or (2) they withdraw consent. Discontinuation because of adverse events will be at either the request of the participant or the discretion of the investigator(s).

#### Interventions

Participants will receive either standard specialist-based follow-up care (usual follow-up care) or shared follow-up care between specialist and PCP (shared care intervention) for a period of 12 months.

#### Control group

Patients in the control group will receive usual care in accordance with hospital practice. Usual follow-up care occurs at 3-monthly intervals during the first year following the end of treatment and includes patient history, physical examination, blood test for carcinoembryonic antigen, and computed tomographic scan at 12 months if recommended by the patient’s treating specialist [34, 35, 70].

#### Intervention group

Patients in the intervention group will receive shared care between specialist and PCP. The shared care intervention will replace two specialist appointments at 3 and 9 months with PCP appointments and add an additional PCP appointment 2 to 6 weeks following the end of treatment to re-establish contact and discuss follow-up care. At baseline, participants allocated to shared care will receive additional resources, including a tailored survivorship care plan, a ‘Living Well after Cancer’ booklet [71] and a DVD titled ‘Just Take It Day to Day’ [72]. A common issues and concerns checklist will also be administered prior to PCP clinic attendance to assist with identification of individual needs. The survivorship care plan will be prepared by the research team and approved by the treating specialist, and it will include diagnosis, treatment history, details about additional hospital services received and information about common issues experienced by CRC survivors, and information about staying well and available community services. The PCP will receive a copy of the survivorship care plan and management guidelines detailing common issues experienced by CRC survivors and how to manage these, as well as how best to contact the specialist treating team for advice or if recurrence is suspected. Both patients and PCPs will receive a reminder letter about upcoming follow-up appointments, with PCPs further reminded to provide information on patient progress and have pathology results copied to specialist.

#### Outcomes

The primary outcome will be overall QoL at 12 months using the European Organisation for Research and Treatment of Cancer core questionnaire (EORTC QLQ-C30) [73]. Secondary outcomes include individual aspects of QoL, unmet needs, continuity of care and satisfaction.

*Individual aspects of QoL:* The EORTC QLQ-C30 functional and symptom scales and the CRC module (EORTC QLQ-CR29) [74] collectively assess specific symptoms such as fatigue, anxiety and pain as well as function on several domains, including physical, role, emotional, cognitive, social, sexual, urinary and bowel.

*Survivors’ unmet needs:* The Short-Form Survivor Unmet Needs Survey [75] is the brief version of the Survivor Unmet Needs Survey 89-item scale. It provides a measure of cancer survivors’ unmet needs, using 30 items across 5 domains: emotional health (8 items), access and continuity of care (6 items), relationships (5 items), financial concerns (8 items) and information (3 items). Each item is scored from 0 (no unmet need) to 4 (very high unmet need) [75].

*Continuity of care:* The Picker Ambulatory Oncology survey comprises eight items that assess patient experience of oncology care [76]. Three items are scored ‘yes completely’, ‘yes somewhat’ or ‘no’ and five items are scored ‘never’, ‘sometimes’, ‘usually’ or ‘always’. Each of the items is fractioned to the number of positive and negative responses. A total score is derived from these positive and negative responses [76].

*Satisfaction:* The Patient Satisfaction Questionnaire short form, derived from the 50-item Patient Satisfaction Questionnaire, comprises 18 items assessing satisfaction [77]. Seven subscales are used: general satisfaction (items 3 and 17), technical quality (items 2, 4, 6 and 14), interpersonal manner (items 10 and 11), communication (items 1 and 13), financial aspects (items 5 and 7), time spent with doctor (items 12 and 15), and accessibility and convenience (items 8, 9, 16 and 18). Each item is scored on a 5-point Likert scale ranging from ‘strongly agree’ to ‘strongly disagree’. Agreement on some scales indicates satisfaction, whereas agreement on other scales reflects dissatisfaction. All items are scored such that high scores reflect satisfaction with medical care.

*Demographics and clinical variables:* A range of demographic and clinical information will be collected for each patient, including age, sex, language spoken at home, living arrangements, postcode, occupation, work status, level of income, diagnosis and stage of disease, and treatment type.

*Health care resource use:* Patients will be consented for access to data on medical service use via Medicare (Australia’s publicly funded universal health care system) from the Commonwealth Department of Human Services. This will provide information on the type, frequency and costs associated with medical service use by participants. Information on hospital service use will be sourced from hospital records on the basis of occurrence of events and costed using Australian Refined Diagnosis Related Groups.

*Recurrence:* To determine recurrence, participants are asked to provide an indication that disease recurrence is suspected; these questions were developed by Cancer Experiences Research, Peter MacCallum Cancer Centre.

*Fidelity:* There are two components to the fidelity section. First, participants are asked to respond whether additional specialist/PCP appointments were scheduled during the follow-up period. Second, there are eight questions that pertain to what participants remember receiving (e.g., survivorship care plan, DVD) as part of the intervention. Questions pertaining to these variables have been sourced from our previous follow-up care survey [27].

**Fig. 2:**
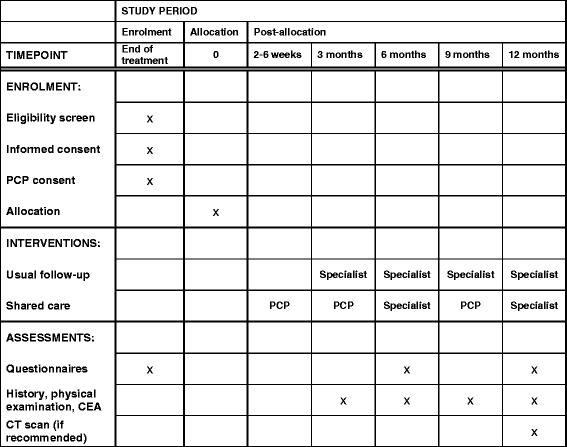
Schedule of appointments and assessment. *CEA* Carcinoembryonic antigen, *CT* Computed tomography, *PCP* Primary care physician

*Participant time line:* The assessment and appointment schedule is detailed in Fig. 2.

#### Sample size

A sample size of 100 patients (50 in each arm) will allow estimation of key parameters with adequate precision to determine the appropriateness of expansion to a definitive phase III trial. The 95% CI for a retention rate of 90% for a study of this size would range from 82% to 95%. A retention rate below 80% would constitute grounds for modifying aspects of the study prior to expansion to a definitive phase III trial. Furthermore, the 95% CIs for the mean difference between the two groups on patient-reported outcome measures would extend no further than ±0.5 SD (given a retention rate of 90%). This level of precision corresponds to what has been proposed as a minimal clinically important difference for health-related QoL measures [78]. Evidence of a substantial detriment associated with shared care as measured by a reduction of 0.6 (or worse) on a patient-reported outcome measure would be detected with 80% power (at the 2.5% one-sided level of significance). A statistically significant negative finding on the global QoL scale from the EORTC QLQ-C30 would constitute grounds for abandoning expansion to a definitive phase III trial.

#### Recruitment

A research team will be appointed at each site. The research team will identify and screen potentially eligible patients from outpatient clinic lists as well as chemotherapy, surgery and radiotherapy lists, with the assistance of clinicians. Eligibility will be confirmed with the treating clinician prior to approach to clarify details from medical records and to ensure the clinician is aware of the patient’s involvement with the study.

Once it has been established that a patient is potentially eligible for the trial, the research team will approach the patient and invite him or her to participate (Additional files 2 and 3). Eligible and consenting patients will complete baseline measures prior to randomisation. The patient’s preferred PCP will be contacted to confirm willingness to be involved in the trial, should the patient be randomised to the intervention arm. An opt-out approach will be used. If the PCP returns the form noting that they prefer not to participate in the study, no further contact will be made. If the PCP contacts the research team stating they would like to take part, or if the form is not returned within 1 week, consent to take part in the study will be implied.

Patients who decline to participate will be asked for their verbal consent to collect basic demographic and clinical information from their records to examine potential recruitment bias. Reasons for refusal will be recorded.

### Assignment of interventions

Trial participants will be randomised to receive either shared care or usual care using a 1:1 ratio following completion of baseline measures. The randomisation sequence will be based on a minimisation scheme with stratification for site (Peter MacCallum Cancer Centre, Royal Melbourne Hospital, Western Health, St Vincent’s Hospital and Austin Hospital). The randomisation sequence will be computer-generated by the research team at Peter MacCallum Cancer Centre using a centralised randomisation database. The allocation sequence will be concealed within a management system (Microsoft Access database; Microsoft, Redmond, WA, USA) managed by a data manager who is independent of the day-to-day conduct of the trial.

### Data collection, management and analysis

#### Data collection methods

The outcome measures will be taken at the end of treatment (baseline) and at 6 and 12 months. Recurrence will be collected at 6 and 12 months with fidelity measures being collected at 12 months. Arrangements of appointments will be self-managed by the participant throughout the trial period. Adherence to follow-up appointments will be collected through Medicare data at the end of the patient’s participation in the trial.

#### Statistical methods

Acceptability of the intervention, randomisation, outcome measures and study processes will be based on a comparison between the expected and observed (1) recruitment rate and (2) proportion of participants completing the study requirements. The planned recruitment rate will be evaluated against the actual recruitment rate in a descriptive fashion (e.g., as a line graph). A point estimate and 95% CI for the proportion completing the study requirements will be calculated and evaluated against a benchmark of 80%. Statistical evidence that the completion probability was inconsistent with the 80% target or that actual recruitment fell appreciably below expectations would constitute grounds for modifying aspects of the study prior to expansion to a definitive phase III trial. The effect of shared care compared with usual care on scales from the patient-reported outcome measures will be quantified by applying a mixed model for repeated measures approach to the data collected. Point estimates of effect on these scales will be presented with 95% CIs. Statistical evidence of a negative impact on the global QoL scale would constitute grounds for abandoning expansion to a definitive phase III trial. This would occur if the two-sided 95% CI for the treatment effect favoured standard care and excluded 0. (Such a result is equivalent to obtaining a significant *p* value at the one-sided 2.5% level of significance.)

Costs and outcomes between usual care and shared care will be compared on the basis of resources required for the delivery of care and the use of medical services by patients. The comparison of outcomes will be focussed on the difference in the proportion of patients with unmet needs allowing an indicative assessment of the cost per additional patient whose needs are met by shared care compared with usual care. Results for the EORTC QLQ-C30 will be converted to preference-based measures of QoL (the EORTC Quality of Life Utility Measure–Core 10 dimensions) [79] for use in estimating quality-adjusted life-years (QALYs). With these data, a potential difference in costs per QALY between usual care and shared care will be explored in a model-based analysis. Differences in cost data will be subject to non-parametric testing, with appropriate sensitivity analyses conducted of comparisons of costs and outcomes [80].

### Monitoring

#### Data monitoring

The study has received ethics approval from the human research ethics committee of Peter MacCallum Cancer Centre (HREC/16/PMCC/89). No significant risks to participants are anticipated. Because the study is unblinded without drug intervention, an independent data and safety monitoring committee is unnecessary; however, the trial management committee will review the recruitment rate, the retention rate, and the data completion rate on an ongoing basis. The trial management committee includes CRC medical and surgical oncologists, PCPs, a behavioural scientist, a statistician, a health economist and a consumer.

#### Safety

Any adverse or unexpected outcomes which occur as a result of the trial will be documented and copies provided to site investigators and the principal investigator within 24 h. The principal investigator will proceed to report any such adverse event to the human research ethics committee.

## Discussion

There is growing interest in post-treatment care in the clinical and research setting, with recognition that current service provision is both inadequate in meeting the needs of survivors and unsustainable, given the growing number of survivors and limited health workforce [32, 37]. It is internationally recognised that models of care are needed which are responsive to the needs of survivors and caregivers and representing more efficient use of limited health care resources [32, 51, 52, 81].

Numerous studies show that survivors of CRC have persisting symptoms, impaired QoL and unmet needs [3, 4, 9–12, 14–16, 18, 20, 21, 26, 28]. They also miss out on health promotion and disease prevention opportunities and appropriate management of comorbid illness [33, 36]. To our knowledge, this is the first study of shared care for people with CRC internationally. Very few studies have addressed survivorship care for CRC survivors. The present clinical trial considers a more comprehensive view of life after cancer, with a focus not just on disease and cancer recurrence but also on dealing with the consequences of cancer and promoting optimal health and well-being [34]. The aim of the intervention is to comprehensively assess needs and link people to necessary multidisciplinary care. The intervention will provide clarity for patients regarding their follow-up and give patients and PCPs the information that they need. Formalising shared care improves communication for survivors, carers and health professionals and limits the underuse and overuse of tests and services [59, 82].

Researchers in a similar study seek to assess QoL in patients with CRC and evaluate an intervention targeted at QoL deficits [83]. This study is based on a similar successful approach for breast cancer survivors [84].

A possible limitation in the SCORE design is differential participation by both CRC survivors and PCPs. We will monitor characteristics of participants and non-participants. Shared care approaches may not be preferred (or appropriate) for all people. In our previous ProCare study, of 84 patients who met eligibility criteria but were not enrolled, only 9 were due to a PCP’s decision to not participate [55].

SCORE considers important outcomes that will remain relevant in the future, including costs, health and well-being, cancer outcomes, management of consequences, illness prevention and chronic disease management. If successful, the findings derived from this study would be transferable to other groups of cancer survivors. The study may also lead to exploration of modifications of shared care protocols: less frequent reviews, remote monitoring or care led by nursing or allied health professionals. It is also likely that demonstration of the impact of shared care in one cancer survivorship setting will lead to exploration and adoption of the model with other groups of cancer survivors.

### Trial status

Currently 16 patients have been recruited.

## Additional files

Additional file 1: Standard Protocol Items: Recommendations for Interventional Trials (SPIRIT) 2013 checklist: recommended items to address in a clinical trial protocol and related documents. (DOC 120 kb)

Additional file 2: Participant information sheet/consent form. (DOCX 55 kb)

Additional file 3: Medicare consent form. (DOCX 33 kb)

## Abbreviations

CEA: Carcinoembryonic antigen
CCM: Chronic Care Model
CRC: Colorectal cancer
CT: Computed tomography
EORTC QLQ-C30: European Organisation for Research and Treatment of Cancer core questionnaire
EOT: End of treatment
IOM: Institute of Medicine
PCP: Primary care physician
QALY: Quality-adjusted life-year
RCT: Randomised controlled trial
SCORE: Shared care of Colorectal cancer survivors intervention
