# Supplementary material for: SCORE: Shared care of Colorectal cancer survivors: protocol for a randomised controlled trial
Source: Trials. 2017 Oct 30;18:506. doi: 10.1186/s13063-017-2245-4 (PMC5663101; doi:10.1186/s13063-017-2245-4)
Supplement: Supplementary file 2 — Participant information sheet/consent form. (DOCX 55 kb) [file 13063_2017_2245_MOESM2_ESM.docx]

**Participant Information Sheet/Consent Form**

**Health/Social Science Research** - *Adult providing own consent*

| **Title** | Shared care of colorectal cancer survivors - A  randomised controlled trial of hospital-based follow up versus shared hospital / community follow up for survivors of colorectal cancer |
| --- | --- |
| **Short Title** | SCORE |
| **Protocol Number** | 16/72 |
| **Project Sponsor** | Peter MacCallum Cancer Centre |
| **Coordinating Principal Investigator/ Principal Investigator** | Associate Professor Michael Jefford |
| **Location** | <insert> |

**Part 1 What does my participation involve?**

1. **Introduction**

You are invited to take part in this research project, which is called SCORE. We are conducting this research to gain a better understanding of how shared care might benefit patients who are being followed up after completing treatment for colorectal cancer. You have been invited because you have had a diagnosis of colorectal cancer and are due to complete treatment or have completed treatment within the last 3 months.

This Participant Information Sheet/Consent Form tells you about the research project. It explains the processes involved with taking part. Knowing what is involved will help you decide if you want to take part in the research.

Please read this information carefully. Ask questions about anything that you don’t understand or want to know more about. Before deciding whether or not to take part, you might want to talk about it with a relative, friend or local health worker.

Participation in this research is voluntary. If you don’t wish to take part, you don’t have to.

If you decide you want to take part in the research project, you will be asked to sign the consent section. By signing it you are telling us that you:

• Understand what you have read

• Consent to take part in the research project

• Consent to be involved in the research described

• Consent to the use of your personal and health information as described.

You will be given a copy of this Participant Information and Consent Form to keep.

**2 What is the purpose of this research?**

We are conducting a research study that is looking at a new way to provide follow-up care for patients who have been treated for colorectal cancer. Even when surgery and/ or radiotherapy and/or chemotherapy treatment has been successful, there is a chance that the cancer can return. Regular follow-up, including check-ups and tests, aims to detect any sign of the cancer early, when it can be treated. Follow-up after treatment for cancer also allows you and your doctors to keep an eye on your health and well-being, including any symptoms or side-effects you may be experiencing.

In Australia, follow-up after treatment for colorectal cancer is usually managed by the specialist doctor who provided your cancer treatment and usually involves regular hospital visits, which include physical examinations.

We are interested in involving general practitioners (GPs) in some of the routine follow-up visits for patients who have been treated for colorectal cancer. This is to assess whether there are benefits to patients and doctors if GPs and specialists work together to provide follow-up care for patients who have been treated for colorectal cancer. In this study we will compare follow-up with specialists (usual care) to shared follow-up involving specialists and GPs (shared care) and look at the benefits. In the past, similar research studies have shown different models of follow-up care to be satisfactory, although there has never been a study of shared care follow-up for patients treated for colorectal cancer.

Approximately 100 men and women from Victoria (from the Peter MacCallum Cancer Centre, the Royal Melbourne Hospital, St Vincent’s Hospital, the Western Hospital and the Austin Hospital in Melbourne) are being invited to participate in this study. You have been selected to participate in this study because you have recently received radiotherapy and/ or surgery and/or chemotherapy for colorectal cancer. Your doctor believes that you should now start routine follow-up and that the follow-up planned as part of this study may be suitable for you. This study will compare two different types of follow-up care; follow-up with your specialist (usual care) or follow-up with your specialist and GP (shared care).

This research is being led by the researcher, Associate Professor Michael Jefford. The research has been funded by the Victorian Cancer Agency.

**3 What does participation in this research involve?**

You have been invited to participate because we have already checked to see if you are suitable to join the study and we have also contacted your regular GP who has agreed to participate in the study if you also decide to participate.

*If you decide you would like to participate in this study:*

1) You will be asked to sign the Participant Consent Forms

2) You will be asked to give permission for the researchers conducting this research project to access your relevant medical records at this hospital and any other hospitals or medical facilities that you attend in relation to this condition. We will collect this information after you enter the research project.

3) You will be asked to complete questionnaires to provide information about yourself, your diagnosis, your physical and emotional state, your health related needs and how your illness is affecting your functioning as a person with colorectal cancer. You will be asked to complete these questionnaires when you consent to participate, and then again 6 and 12 months later. You will be given the option of having these questionnaires mailed to you or completing these online and they will take about 20 minutes to complete.

4) You will be asked to fill out a consent form authorising the study access to your complete Medicare data as outlined on the back of the consent form. Medicare collects information on your medical visits and procedures, and the associated costs. The consent form is sent securely to the Department of Human Services who holds this information confidentially.

Once you have signed the consent form you will be asked to complete the baseline questionnaire and then you will be randomised into one of the study groups described below. Randomisation means that you are put into a group by chance, like the toss of a coin. A computer program will choose which group you are allocated to. Neither you nor your doctor can choose the group you will be in. You will have an equal chance of being placed in either group. You will be told which group you are placed in and what type of follow-up care you will receive.

**Usual care group (this is called the control arm)**

If you are in this group you will receive regular follow-up care with your specialist at your hospital every 3 months. You will be asked to complete 2 more follow-up questionnaires, 6 months and 12 months after you have completed your treatment. You will be given the option of having these questionnaires mailed to you or completing these online and they will take about 20 minutes to complete.

**Trial shared care group (this is called the intervention arm)**

If you are in this group you will receive follow-up care with both your hospital specialist and GP. You will see your hospital specialist at 6 months and 12 months, but your hospital specialist appointments at 3 months and 9 months will be replaced with GP appointments. You will also have an extra appointment with your GP shortly after the end of your treatment to discuss your follow-up care. You will receive a reminder letter and an SMS about two weeks prior to the due date of these appointments. If you are in this group you will also be asked to complete 2 more follow-up questionnaires, 6 months and 12 months after completing your treatment. You will be given the option of having these questionnaires mailed to you or completing these online and they will take about 20 minutes to complete.

|  | Recruitment to study | 2 weeks after recruitment | 3 months | 6 months | 9 months | 12 months |
| --- | --- | --- | --- | --- | --- | --- |
| Usual care arm |  |  | Hospital | Hospital | Hospital | Hospital |
| Trial shared-care arm |  | GP | GP | Hospital | GP | Hospital |
| Participant Questionnaires | ✔ |  | ✔ | ✔ |  | ✔ |

If you are in the trial care shared group you will receive:

1) A follow-up care plan that is prepared for you, which summarises information about your colorectal cancer diagnosis and treatment, symptoms to watch for, and your follow-up care. A member of the research team will call you to review your follow-up care plan with you, to make sure it is accurate and covers any issues or concerns that are specific to you. Your hospital doctor will also review the accuracy of the survivorship care plan.

2) The ‘Living well after cancer’ booklet produced by the Cancer Council and the Australian Cancer Survivorship Centre DVD; ‘Just take it day to day’. These materials include general information about post-treatment issues, the impact of a healthy lifestyle, guidelines about surveillance and screening for other cancers.

3) A common concerns and issues check list will be mailed to you prior to each GP appointment. This list is to be completed prior to your scheduled GP visit and is a list of common issues faced by people with colorectal cancer. This list is to help you and your GP identify any issues that you might like to discuss during your follow up appointment.

4) Your GP will also receive GP management guidelines, which include the same summary of your colorectal cancer diagnosis and treatments that you will receive with your survivorship care plan. These guidelines will also include details about common issues survivors of colorectal cancer may experience, information on cancer spread and recurrence, the best way to contact your hospital treating team and information on surveillance and also prevention.

The types of follow-up check-ups and tests will be similar in both groups.

The entire study will be running for 3 years; however we are only asking you to be involved for the 12 months following their treatment.

At the conclusion of the study a plain language summary of the project results will be made available upon request.

Participating in this study may also mean that you are required to visit your GP more often than you would normally need to. Although GP visits planned as part of this study will be eligible for Medicare reimbursement you may be required to pay the gap not covered by Medicare.

**4 Other relevant information about the research project**

Approximately 100 men and women, 50 in usual care and 50 in the intervention shared care, are being invited to participate in this study. Participants are from 5 hospitals in Victoria, Peter MacCallum Cancer Centre, the Royal Melbourne Hospital, St Vincent’s Hospital, the Western Hospital and the Austin Hospital.

**5 Do I have to take part in this research project?**

Participation in this study is voluntary. It is completely up to you whether or not you wish to participate. If you agree to participate in this study, you will be asked to attend the follow-up visits and complete the study questionnaires as outlined above. If you decide not to participate in the study you will receive usual care follow-up with your specialist. Your choice not to participate will not affect the treatment you receive now or in the future. Whatever your decision, it will not affect your relationship with the staff caring for you.

You should be aware that data collected up to the time you withdraw will form part of the research project results. If you do not want your data to be included, you must tell the researchers when you withdraw from the research project.

**6 What are the possible benefits of taking part?**

We cannot guarantee or promise that you will receive any benefits from this research; however, possible benefits may include a more satisfactory type of follow-up care for some participants. We hope the information learned from this study will benefit other patients with colorectal cancer in the future.

**7 What are the possible risks and disadvantages of taking part?**

*Follow-up appointments*

It is possible that participation in this study may mean that you are required to visit your specialist or GP more often than you would normally need to if you were not participating in the study. It is also possible that patients in the shared care group may be required to make some up-front payments to see their GP. Although GP visits planned as part of this study will be eligible for Medicare reimbursement as per usual there may be a GAP payment.

*Questionnaires*

You may feel that some of the questions we ask are stressful or upsetting. If you do not wish to answer a question, you may skip it and go to the next question, or you may stop immediately. If you become upset or distressed as a result of your participation in the research project, the research team will be able to arrange for counselling or other appropriate support. Any counselling or support will be provided by qualified staff who are not members of the research team. This counselling will be provided free of charge.

**8 What if I withdraw from this research project?**

If you do consent to participate, you may withdraw at any time. If you decide to withdraw from the project, please notify a member of the research team before you withdraw. A member of the research team will inform you if there are any special requirements linked to withdrawing. If you do withdraw, you will be asked to complete and sign a ‘Withdrawal of Consent’ form; this will be provided to you by the research team.

If you decide to leave the research project, the researchers will not collect additional personal information from you, although personal information already collected will be retained to ensure that the results of the research project can be measured properly and to comply with law. You should be aware that data collected up to the time you withdraw will form part of the research project results. If you do not want your data to be included, you must tell the researchers when you withdraw from the research project.

**9 Could this research project be stopped unexpectedly?**

All types of research depend on the availability of adequate research funds. The researchers involved in this project are highly committed and will do their best to secure the necessary funding.

**10 What happens when the research project ends?**

This study is looking at follow-up care options for the first 12 months after the end of treatment for colorectal cancer, but follow-up care for colorectal cancer should continue beyond the end of the study period. At the end of the study, you should discuss your ongoing follow-up care plan with your doctor(s) and decide what follow-up care is best for you.

If you are interested in looking at the results of the study, please inform one of the project staff. We can then organise to provide you with a summary of results after the research study has been completed.

**Part 2 How is the research project being conducted?**

**11 What will happen to information about me?**

By signing the consent form you consent to the research team collecting and using personal information about you for the research project. Any information obtained in connection with this research project that can identify you will remain confidential.

- All your questionnaires will be stored securely in such a way that they cannot immediately be identified as having come from you.
- Any identifying information (your full name, address, etc.) will be stored separately from the information you provide. Access to this identifying information is restricted to a small number of members of the project team.
- Information about you may be obtained from your health records held at this and other health organisations for the purpose of the study. By signing the consent form you agree to the project team accessing health records if they are relevant to your participation in this project.
- In accordance with relevant Australian and/or Victorian privacy and other relevant laws, you have the right to request access to the information about you that is collected and stored by the project team. You also have the right to request that any information with which you disagree be corrected. Please inform the project team member named at the end of this document if you would like to access your information.
- The information collected about you as part of this study will be kept in locked filing cabinets and on computer files that are password protected. Data will be kept at Peter MacCallum Cancer Centre. Data will be retained for a minimum of 15 years from the date of publication of the results.
- The Medicare data that is collected as part of this study will be stored securely at Peter MacCallum Cancer Centre. This will be destroyed after 7 years from publication of the final project report or after 10 years from the date the information is supplied by the Department of Human Services, whichever is sooner.
- It is anticipated that the results of this research project will be published and/or presented in a variety of forums. In any publication and/or presentation, information will be provided in such a way that you cannot be identified, except with your express permission.
- Your non-identifiable data, excluding any Medicare data collected, may also be used in future studies and research. Any future research accessing your data would have to be approved by an ethics committee and the SCORE steering committee. Any information obtained for the purpose of this research project, or the future research that can identify you will be treated as confidential and securely stored. It will be disclosed only with your permission, or as required by law. Information collected from Medicare will not be used in any future studies and research.

**12 Complaints and compensation**

If you suffer any distress or psychological injury as a result of your participation in this project, you should contact the project team as soon as possible. You will be assisted with arranging appropriate treatment and support free of charge.

**13 Who is organising and funding the research?**

This research project is being conducted by Associate Professor Michael Jefford at Peter MacCallum Cancer Centre. It is being funded by the Victorian Cancer Agency.

No member of the research team will receive a personal financial benefit from your involvement in this research project (other than their ordinary wages).

**14 Who has reviewed the research project?**

All research in Australia involving humans is reviewed by an independent group of people called a Human Research Ethics Committee (HREC). The ethical aspects of this research project have been approved by the HREC of Peter MacCallum Cancer Centre.

This project will be carried out according to the *National Statement on Ethical Conduct in Human Research (2007)*. This statement has been developed to protect the interests of people who agree to participate in human research studies.

**15 Further information and who to contact**

The person you may need to contact will depend on the nature of your query. If you want any further information concerning this project or if you have any problems which may be related to your involvement in the project, you can contact any of the following people:

**Research contact person**

| Name | Paula Rodger |
| --- | --- |
| Position | Project Coordinator |
| Telephone | 8559 5910 |
| Email | paula.rodger@petermac.org |

For matters relating to research at the site at which you are participating, the details of the local site complaints person are:

**Complaints contact person**

| Position | <insert> |
| --- | --- |
| Telephone | <insert> |
| Email | <insert> |

If you have any complaints about any aspect of the project, the way it is being conducted or any questions about being a research participant in general, then you may contact:

| Reviewing HREC name | Peter MacCallum Cancer Centre Ethics Committee |
| --- | --- |
| HREC Executive Officer | Ethics Coordinator |
| Telephone | 03 8559 7540 |
| Email | ethics@petermac.org |

**Reviewing HREC approving this research** **and HREC Executive Officer detail**

**Consent Form -** *Adult providing own consent*

| **Title** | Shared care of colorectal cancer survivors – A randomised controlled trial of hospital-based follow-up versus shared hospital / community follow up for survivors of colorectal cancer |
| --- | --- |
| **Short Title** | SCORE |
| **Protocol Number** | *16/72* |
| **Principal Investigator** | Associate Professor Michael Jefford |
| **Location** | <insert> |

**Declaration by Participant**

I have read the Participant Information Sheet or someone has read it to me in a language that I understand.

I understand the purposes, procedures and risks of the research described in the project.

I have had an opportunity to ask questions and I am satisfied with the answers I have received.

I freely agree to participate in this research project as described and understand that I am free to withdraw at any time during the project without affecting my future care.

I understand that I will be given a signed copy of this document to keep.

🞎 I would like to complete my questionnaires online

Email: ______________________________________

|  | | | | | | | |
| --- | --- | --- | --- | --- | --- | --- | --- |
|  | Name of Participant (please print) | |  | |  |  |  |
|  | | | | | | | |
|  | Signature |  | | Date | |  |  |
|  | | | | | | | |

**Declaration by Researcher^†^**

I have given a verbal explanation of the research project, its procedures and risks and I believe that the participant has understood that explanation.

|  | | | | | | |
| --- | --- | --- | --- | --- | --- | --- |
|  | Name of Researcher^†^ (please print) | |  | | |  |
|  | | | | | |  |
|  | Signature |  | | Date |  |  |
|  | | | | | | |

^†^ An appropriately qualified member of the research team must provide the explanation of, and information concerning, the research project.

Note: All parties signing the consent section must date their own signature.

**Form for Withdrawal of Participation -** *Adult providing own consent*

| **Title** | Shared care of colorectal cancer survivors – A randomised controlled trial of hospital-based follow-up versus shared hospital / community follow up for survivors of colorectal cancer |
| --- | --- |
| **Short Title** | SCORE |
| Protocol Number | 16/72 |
| **Principal Investigator** | Associate Professor Michael Jefford |
| **Location** | <insert> |

**Declaration by Participant**

I wish to withdraw from participation in the above research project and understand that such withdrawal will not affect my routine care, or my relationships with the researchers or treating hospital.

|  | | | | | | | |
| --- | --- | --- | --- | --- | --- | --- | --- |
|  | Name of Participant (please print) | |  | |  |  |  |
|  | | | | | | | |
|  | Signature |  | | Date | |  |  |
|  | | | | | | | |

In the event that the participant’s decision to withdraw is communicated verbally, the Senior Researcher must provide a description of the circumstances below.

|  |
| --- |

**Declaration by Researcher^†^**

I have given a verbal explanation of the implications of withdrawal from the research project and I believe that the participant has understood that explanation.

|  | | | | | | |
| --- | --- | --- | --- | --- | --- | --- |
|  | Name of Researcher (please print) | |  | | |  |
|  | | | | | |  |
|  | Signature |  | | Date |  |  |
|  | | | | | | |

^†^ An appropriately qualified member of the research team must provide information concerning withdrawal from the research project.

Note: All parties signing the consent section must date their own signature.
