# Supplementary material for: SCORE: Shared care of Colorectal cancer survivors: protocol for a randomised controlled trial
Source: Trials. 2017 Oct 30;18:506. doi: 10.1186/s13063-017-2245-4 (PMC5663101; doi:10.1186/s13063-017-2245-4)
Supplement: Supplementary file 3 — Medicare consent form. (DOCX 33 kb) [file 13063_2017_2245_MOESM3_ESM.docx]

### Participant ID:

***PARTICIPANT CONSENT FORM***

Consent to release of Medicare and/or Pharmaceutical Benefits Scheme (PBS) claims information for the purposes of Score – Shared care of colorectal cancer survivors Study

**Important Information**

Complete this form to request the release of personal Medicare claims information and/or PBS claims information to Score – Shared care of colorectal cancer survivors Study.

Any changes to this form must be initialled by the signatory. Incomplete forms may result in the study not being provided with your information.

By signing this form, I acknowledge that I have been fully informed and have been provided with information about this study. I have been given an opportunity to ask questions and understand the possibilities of disclosures of my personal information.

**PARTICIPANT DETAILS**

**1.** Mr □ Mrs □ Miss □ Ms □ Other

Family name: ________________________________ First given name: _________________________

Other given name (s): __________________________

Date of birth: DD/MM/YYYY

**2.** Medicare card number: ______________________

**3.** Permanent address: _____________________________________________________________

Postal address (if different to above): ________________________________________________

**AUTHORISATION**

**4.** I authorise the Department of Human Services to provide my:

Medicare claims history

for the period* DD/MM/YYYY to: DD/MM/YYYY to the Score – Shared care of colorectal cancer survivors Study.

*Note: The Department of Human Services can only extract 4.5 years of data (prior to the date of extraction), The consent period above may result in multiple extractions.

**DECLARATION**

I declare that the information on this form is true and correct.

**5.** Signed: ______________________ (participant’s signature) Dated: DD/MM/YYYY **OR**

**6.** Signed by ____________________ (full name) __________________ (signature) on behalf of participant

Dated: DD/MM/YYYY

Power of attorney** Guardianship order**

** Please attach supporting evidence

**APP 5 – PRIVACY NOTICE**

Your personal information is protected by law, including the Privacy Act 1988, and is collected by the Australian Government Department of Human Services. The collection of your personal information by the department is necessary for administering requests for statistical and other data.

Your information may be used by the department or given to other parties for the purposes of research, investigation or where you have agreed or it is required or authorised by law.

You can get more information about the way in which the Department of Human Services will manage your personal information, including our privacy policy at humanservices.gov.au/privacy or by requesting a copy from the department.

**Power of attorney** – A power of attorney is a document that appoints a person to act on behalf of another person who grants that power. In particular, an enduring power of attorney allows the appointed person to act on behalf of another person even when that person has become mentally incapacitated. The powers under a power of attorney may be unlimited or limited to specific acts.

**Guardianship order** – A Guardianship order is an order made by a Guardianship Board/Tribunal that appoints a guardian to make decisions for another person. A Guardianship order may be expressed broadly or limited to particular aspects of the care of another person.

**A sample of the information that may be included in your Medicare claims history:**

| **Date of service** | **Item number** | **Item description** | **Provider charge** | **Schedule Fee** | **Benefit paid** | **Patient out of pocket** |
| --- | --- | --- | --- | --- | --- | --- |
| 20/04/09 | 00023 | Level B consultation | $38.30 | $34.30 | $34.30 | $4.00 |
| 22/06/09 | 11700 | ECG | $29.50 | $29.50 | $29.50 |  |

| **Scrambled rendering Provider number*** | **Rendering Provider postcode** | **Ordering Provider postcode** | **Hospital**  **indicator** | **Item category** |
| --- | --- | --- | --- | --- |
| 999999A | 2300 |  | N | 1 |
| 999999A | 2300 | 2302 | N | 2 |

* Scrambled Provider number refers to a unique scrambled provider number identifying the doctor who provided/referred the service. Generally, each individual provider number will be scrambled and the identity of that provider will not be disclosed.
